# Supplementary material for: The role of interspecies recombination in the evolution of antibiotic-resistant pneumococci
Source: eLife. 2021 Jul 14;10:e67113. doi: 10.7554/eLife.67113 (PMC8321556; doi:10.7554/eLife.67113)
Supplement: Figure 9—source data 1. — The species, ENA accession codes, and frequencies of the matches to the 500 bp region upstream of the 66 Tn1207.1-type element insertions into tag that were reconstructed as having occurred within inferred homologous recombination events. [file elife-67113-fig9-data1.docx]

| Species | ENA accession | Frequency |
| --- | --- | --- |
| *S. mitis* | AYRR00000000 | 56 |
| *S. mitis* | AYRS00000000 | 2 |
| *S. mitis* | JPFW00000000 | 2 |
| *S. mitis* | AEDU00000000 | 1 |
| *S. mitis* | AEDV00000000 | 1 |
| *S. mitis* | AJJL00000000 | 1 |
| *S. mitis* | AQTU00000000 | 1 |
| *S. pseudopneumoniae* | AYRN00000000 | 1 |
| *S. pneumoniae* | D39 | 1 |

**Figure 9—source 1: Closest species match to 500 bp region upstream of *tag* disrupting Tn*1207.1* insertion.** The species, ENA accession codes, and frequencies of the matches to the 500 bp region upstream of the 66 Tn*1207.1 tag* insertions
