# Supplementary material for: The role of interspecies recombination in the evolution of antibiotic-resistant pneumococci
Source: eLife. 2021 Jul 14;10:e67113. doi: 10.7554/eLife.67113 (PMC8321556; doi:10.7554/eLife.67113)
Supplement: Transparent reporting form [file elife-67113-transrepform.docx]

***eLife’s* transparent reporting form**

We encourage authors to provide detailed information *within their submission* to facilitate the interpretation and replication of experiments. Authors can upload supporting documentation to indicate the use of appropriate reporting guidelines for health-related research (see [EQUATOR Network](http://www.equator-network.org/%20%20%5Ct%20_blank)), life science research (see the [BioSharing Information Resource](https://biosharing.org/)), or the [ARRIVE guidelines](http://www.plosbiology.org/article/info:doi/10.1371/journal.pbio.1000412) for reporting work involving animal research. Where applicable, authors should refer to any relevant reporting standards documents in this form.

If you have any questions, please consult our Journal Policies and/or contact us: [editorial@elifesciences.org](mailto:editorial@elifesciences.org).

**Sample-size estimation**

- You should state whether an appropriate sample size was computed when the study was being designed
- You should state the statistical method of sample size computation and any required assumptions
- If no explicit power analysis was used, you should describe how you decided what sample (replicate) size (number) to use

Please outline where this information can be found within the submission (e.g., sections or figure legends), or explain why this information doesn’t apply to your submission:

**No explicit power analysis was performed; instead, all available samples were used in the analyses. Detailed phylogenetic analysis was only performed on samples which were systematically collected, as outlined in Methods section, “Phylogenetic and phylodynamic analyses”, and within the Results section “Selection for local expansion of macrolide resistant *S. pneumoniae”.***

**Replicates**

- You should report how often each experiment was performed
- You should include a definition of biological versus technical replication
- The data obtained should be provided and sufficient information should be provided to indicate the number of independent biological and/or technical replicates
- If you encountered any outliers, you should describe how these were handled
- Criteria for exclusion/inclusion of data should be clearly stated
- High-throughput sequence data should be uploaded before submission, with a private link for reviewers provided (these are available from both GEO and ArrayExpress)

Please outline where this information can be found within the submission (e.g., sections or figure legends), or explain why this information doesn’t apply to your submission:

**The inclusion and exclusion criteria for whole genome sequencing data are outlined in the Methods section, “Bacterial isolates and DNA sequencing”. All raw and processed sequence data are publicly available, as outlined in this section, with new data available from the European Nucleotide Archive project PRJEB2255.**

**Statistical reporting**

- Statistical analysis methods should be described and justified
- Raw data should be presented in figures whenever informative to do so (typically when N per group is less than 10)
- For each experiment, you should identify the statistical tests used, exact values of N, definitions of center, methods of multiple test correction, and dispersion and precision measures (e.g., mean, median, SD, SEM, confidence intervals; and, for the major substantive results, a measure of effect size (e.g., Pearson's r, Cohen's d)
- Report exact p-values wherever possible alongside the summary statistics and 95% confidence intervals. These should be reported for all key questions and not only when the p-value is less than 0.05.

Please outline where this information can be found within the submission (e.g., sections or figure legends), or explain why this information doesn’t apply to your submission:

(For large datasets, or papers with a very large number of statistical tests, you may upload a single table file with tests, Ns, etc., with reference to sections in the manuscript.)

**These details can be found in the Results sections “Multiple independent acquisitions of resistance genes in *S. pneumoniae”*, *“*Variation in transformation rates and imported sequence properties” and *“*Interspecies origin of MGEs”. All sections use Mann-Whitney tests, and in each case, we report the total number of samples, N; the value of the test statistic, W; and the exact two-tailed *p* value. The raw data for the difference in recombination lengths and SNP density tests are shown in Figures 5 and 8. In other figures, it was not feasible to informatively display the raw data, due to large sample sizes. The phylodynamic statistical tests described in "Selection for local expansion of macrolide resistant *S. pneumoniae*” report the mean value and associated 95% credibility intervals calculated from Monte Carlo Markov chains. These were run for 100 million iterations, with the first 20 million iterations removed as warm-up, to ensure only values sampled from the point at which the chains had converged were used in calculating the reported values.**

**Group allocation**

- Indicate how samples were allocated into experimental groups (in the case of clinical studies, please specify allocation to treatment method); if randomization was used, please also state if restricted randomization was applied
- Indicate if masking was used during group allocation, data collection and/or data analysis

Please outline where this information can be found within the submission (e.g., sections or figure legends), or explain why this information doesn’t apply to your submission:

**No randomization or blinding was used in this study. Sub-analyses on groups were performed based on geographic, phenotypic and phylogenetic information. In particular, a detailed analysis was performed on a clade of isolates systematically collected from Germany in the section entitled, “Selection for local expansion of macrolide resistant *S. pneumoniae*”. This analysis can only be performed once both the epidemiological data, and population structure, were known, and could not be performed on a set of isolates determined prior to the genome sequences being available.**

**Additional data files (“source data”)**

- We encourage you to upload relevant additional data files, such as numerical data that are represented as a graph in a figure, or as a summary table
- Where provided, these should be in the most useful format, and they can be uploaded as “Source data” files linked to a main figure or table
- Include model definition files including the full list of parameters used
- Include code used for data analysis (e.g., R, MatLab)
- Avoid stating that data files are “available upon request”

Please indicate the figures or tables for which source data files have been provided:

**We have made all data freely available where possible; specifically:**

- **The phylogenetic trees and epidemiological data used for Figures 1 and 2 are available for download and analysis from** [**https://microreact.org/project/71AY1wepkqsJ5JT8JgrLpc**](https://microreact.org/project/71AY1wepkqsJ5JT8JgrLpc) **and** [**https://microreact.org/project/kX8fDp8U2Q2H35VW5xo3o**](https://microreact.org/project/kX8fDp8U2Q2H35VW5xo3o)
- **The code used to predict antibiotic resistant phenotypes is available from the Github repository** [**https://github.com/jdaeth274/pbp_tpd_extraction**](https://github.com/jdaeth274/pbp_tpd_extraction)
- **The code used in the insertion site analysis, *pbp* gene origin and *cps* locus switching switching analyses is available from the Github repository** [**https://github.com/jdaeth274/ISA**](https://github.com/jdaeth274/ISA)
- **The source data for main figures is available from FigShare, using the URL** [**https://doi.org/10.6084/m9.figshare.c.5306462.v1**](https://doi.org/10.6084/m9.figshare.c.5306462.v1)
- **The sequences used in this study, along with their assembly accession codes and sample accession codes, are detailed in Table S1.**
